# Association of Tumor Necrosis Factor Alpha, Interleukin 6, and C-Reactive Protein with the Risk of Developing Type 2 Diabetes: A Retrospective Cohort Study of Rural Thais

**DOI:** 10.1155/2019/9051929

**Published:** 2019-08-08

**Authors:** Jirayu Lainampetch, Pornpimol Panprathip, Chanchira Phosat, Noppanath Chumpathat, Pattaneeya Prangthip, Ngamphol Soonthornworasiri, Somchai Puduang, Naruemon Wechjakwen, Karunee Kwanbunjan

**Affiliations:** ^1^Department of Tropical Nutrition and Food Science, Faculty of Tropical Medicine, Mahidol University, Bangkok 10400, Thailand; ^2^Department of Nutrition, Faculty of Public Health, Mahidol University, Bangkok 10400, Thailand; ^3^Faculty of Nursing, Huachiew Chalermprakiet University, Samut Prakan 10540, Thailand; ^4^Department of Tropical Hygiene, Faculty of Tropical Medicine, Mahidol University, Bangkok 10400, Thailand; ^5^Faculty of Public Health, Nakhon Ratchasima Rajabhat University, Nakhon Ratchasima 30000, Thailand

## Abstract

The linkage of obesity, inflammation, and type 2 diabetes mellitus (T2DM) has been extensively investigated for over a decade. However, the association between inflammatory biomarkers, including C-reactive protein (CRP), interleukin 6 (IL-6), and tumor necrosis factor alpha (TNF-*α*), and T2DM is still inconsistent and limited. Thus, this study is aimed at elucidating the association between inflammatory marker levels and the risk of developing T2DM in many aspects. Among 296 subjects enrolled in 2013, 248 non-T2DM subjects who were completely reinvestigated in 2014 and 2015 were included in a 2-year retrospective analysis. Multivariate logistic regression was performed to evaluate the association of baseline inflammatory marker levels and variation with incidence of T2DM. After the 2-year follow-up, 18.6% of total subjects had developed T2DM. The risk of developing T2DM was significantly increased in subjects with a high level of baseline CRP (OR = 4.02, 95% CI: 1.77-9.12, *P* = 0.001), and a stronger impact was found with the combination of high CRP and IL-6 levels (OR = 5.11, 95% CI: 1.27-20.49, *P* = 0.021). One-year inflammatory marker variation analysis also revealed the significant association of elevated TNF-*α* and risk of developing T2DM (OR = 4.88, 95% CI: 1.01-23.49, *P* = 0.048). In conclusion, besides consideration of CRP levels alone, our findings suggested that IL-6 outstandingly plays a contributing role in T2DM progression and elevated TNF-*α* levels over time could be a potential predictor of T2DM.

## 1. Introduction

Diabetes is an important cause of morbidity and mortality that leads the top ten public health burdens in Thailand [1]. According to the recent report in 2017, the prevalence of diabetes was 8.3% among a nationwide adult population, representing over 4 million diabetic cases in Thailand [2]. The northeastern region was estimated to have the highest morbidity rate, especially in those aged 35-60 years [3]. This may be due to the widespread transition from an agricultural to an industrial society in this region that changes lifestyle habits and subsequently contributes to metabolic disease.

C-reactive protein (CRP), a major acute-phase protein, is generally considered as an indicator for low-grade systemic inflammation in chronic diseases such as cancer and cardiovascular disease (CVD) [4]. In regard to T2DM, CRP is also known as an independent predictor of T2DM [5–9]. Previous studies have extensively reported the association of elevated CRP levels with insulin resistance and progression of T2DM [6, 10–12].

High caloric intake with physical inactivity causes excess fat accumulation, indicating as the major risk factor of insulin resistance and type 2 diabetes mellitus (T2DM) [13]. Evidences showed that hypertrophied adipocytes involve with an inflammatory condition [14–18]. Inflammatory cytokines secreted by adipose tissue, such as interleukin 6 (IL-6) and tumor necrosis factor alpha (TNF-*α*), may exert an endocrine effect to promote insulin resistance by interfering with the insulin signalling pathway, leading to the clinical manifestation of T2DM [19, 20].

Clinical findings revealed the role of IL-6 and TNF-*α* in glucose homeostasis and metabolism and also the indirectly possible action on the pancreatic *β*-cell [21, 22]. Furthermore, the presence of these cytokines leads to inflammatory responses with elevated CRP [23–25]. IL-6 and TNF-*α* were suggested as a possible marker for T2DM prediction [26–28]. However, nonstatistically significant results have also been reported [8, 12, 29]. The biological role of these cytokines on T2DM development may still be uncertain.

The linkage between inflammation and T2DM which was found in a cross-sectional study showed difficulty in considering the sequence of events and may not reflect the true risk of disease. A single baseline measurement of CRP, IL-6, and TNF-*α* levels was unable to evaluate the effects of changes over time, and no evidence of interaction between these inflammatory markers was found. We therefore designed a cohort study with different analyses to determine the association between inflammatory marker levels and the risk of developing T2DM among Thai adults in the rural area of the northeastern region where urbanization and industrialization are rapidly growing. Our study was aimed at evaluating the potential of the T2DM predictor in longitudinal analysis.

## 2. Materials and Methods

### 2.1. Study Design and Data Source

A retrospective cohort study on rural Thai was carried out in 12 villages of Sung Noen District, Nakhon Ratchasima Province, from 2013 to 2015. The data set was extracted from 296 participants, consisting of demographic information, dietary intake, anthropometric assessments, and biochemical analysis. The description of subject characteristics, selection criteria, and data collection have been published elsewhere [12].

### 2.2. Study Subjects

This study includes nondiabetic subjects aged 35-66 years who were initially examined in 2013 (baseline) and subsequently reexamined based on identical procedures in 2014 and 2015. After excluding those who identified as T2DM at baseline by fasting blood glucose (FBG), 2-hour blood glucose (2-h BG), and glycated hemoglobin (HbA1c) [30], 248 eligible subjects who completed on reexamination in 2014 and 2015 were entered for longitudinal analysis. The study protocol was approved by the Ethics Committee of the Faculty of Tropical Medicine, Mahidol University (TMEC 18–040).

### 2.3. Criteria of Main Outcome: Incidence of T2DM

Incident cases of T2DM were determined as FBG ≥ 126 mg/dl (7.0 mmol/l), 2 − h BG ≥ 200 mg/dl (11.1 mmol/l), and HbA1c ≥ 6.5% (48 mmol/mol) at reexamination according to the American Diabetes Association (ADA) criteria [30] or with a clinical diagnosis for T2DM during the follow-up period. A total of 30 new cases of T2DM were found in the 2014 reexamination; 16 new cases were found in the 2015 reexamination.

### 2.4. Statistical Analysis

Continuous variables were expressed as median (interquartile range) and the differences were compared by Mann-Whitney *U* test. For categorical variables, data were reported as proportions (%) and compared using chi-squared tests. To investigate the relationship of inflammation and T2DM, univariate and multivariate logistic regressions were used to calculate the odds ratios (ORs) and 95% confidence intervals (CIs) of the baseline inflammatory marker levels for 1-year and 2-year progressions of T2DM. The role of inflammatory marker interaction was examined. Baseline TNF-*α* and IL-6 levels were classified using the tertile as the cut-off point (setting the subjects who were 1^st^ tertile for both TNF-*α* and IL-6 as the low level and those who were 3^rd^ tertile as the high level), and baseline CRP levels were classified by suggested cut-off values [31]. ORs and 95% CIs were estimated for each combination, including two and three of these inflammatory markers. In addition, the association between changes in inflammatory marker levels from baseline (2013) to 2014 and the risk of developing T2DM at 2 years was analyzed. Changes in serum inflammatory marker levels were divided into four categories: low level in both 2013 and 2014, low level in 2013 but high in 2014, high level in 2013 but low in 2014, and high level in both 2013 and 2014. All statistical analyses were performed using SPSS (version 18.0; SPSS, Chicago, IL, USA). A *P* value < 0.05 was considered statistically significant.

## 3. Results

### 3.1. Baseline Characteristics of Study Subjects

A total of 248 diabetes-free individuals composed of 82 males and 166 females with fully completed data collections and laboratory analyses in 2013 and who were completely reexamined in 2014 and 2015 were included. They were divided into two groups according to incidence of T2DM as follows: the nonprogression group: subjects who were diabetes-free throughout the study period, and the progression group: subjects who were identified as T2DM at any reexamination year.  Table 1 displays baseline demographic and anthropometric, biochemical, clinical, and dietary characteristics of the subjects with or without T2DM progression at reexamination. Age of new-onset T2DM participants was rather older (*P* = 0.035), and half of them had a diabetes family history. Blood glucose and insulin resistance results at baseline in the T2DM groups substantially showed greater values than the normal group. Furthermore, their baseline BMI, WC, WHR, VF, DBP, and triglyceride were elevated higher but with lower baseline HDL-C levels. However, dietary intake assessed from a semifood frequency questionnaire (semi-FFQ) showed no statistical difference between the two groups. In regard to inflammatory markers, subjects with T2DM obviously indicated a higher baseline CRP level, but the difference of IL-6 and TNF-*α* levels between groups was not statistically significant.

### 3.2. Relationship of Anthropometric and Metabolic Parameters with Serum Inflammatory Markers at Baseline

Using the Mantel-Haenszel chi-squared trend test, we found that low physical activity subjects (exercise less than 3 times per week) indicated higher proportions of highest IL-6 and TNF-*α* levels than the physically active group (40.2% vs. 28.4%, *P* for trend = 0.014, and 43.3% vs. 26.5%, *P* for trend = 0.006, respectively). Likewise, low physical activity subjects showed a higher CRP level (68.9% vs. 43.3%) compared with individuals who exercise frequently (*P* < 0.001). Furthermore, Spearman's rank correlation coefficients were calculated. Serum CRP correlated significantly with WHR (*r* = 0.213, *P* = 0.001), whereas IL-6 and TNF-*α* only showed a positive trend towards correlation with WHR (*r* = 0.094 and *r* = 0.123, respectively). CRP showed a positive correlation with HOMA-IR (*r* = 0.322, *P* < 0.001) and FBG (*r* = 0.131, *P* = 0.040), and IL-6 positively correlated with FBG (*r* = 0.126, *P* = 0.047). Significant correlations were also considerably found between CRP and other parameters, including BMI (*r* = 0.325), WC (*r* = 0.359), total cholesterol (*r* = 0.132), HDL-C (*r* = −0.177), and LDL-C (*r* = 0.169).

### 3.3. Predictive Effect of Baseline Serum Inflammatory Markers on Blood Glucose Status

A linear regression model was performed to elucidate the metabolic correlation. In a multivariable regression analysis, with FBG, 2-h BG, and HbA1C as dependent variables and age, sex, CRP, IL-6, and TNF-*α* as independent variables, significant associations were shown between FBG and age, sex, CRP, and IL-6 (*F* = 4.759, *P* < 0.001) but not TNF-*α* level. For 2-h BG and HbA1C, CRP showed the strong associations with these indices, implying as a good predictor for blood glucose status specified by FBG, 2-h BG, and HbA1C (*β* = 0.216, *P* = 0.001, and *β* = 0.223, *P* < 0.001, respectively) (Table 2).

### 3.4. The Association between Baseline Serum Inflammatory Marker Levels and the Risk of Developing T2DM

The results of logistic regression for the 2-year T2DM progression are presented in Table 3. Study subjects with CRP levels ≥ 1 mg/dl had approximately a fourfold (OR = 4.20, 95% CI: 1.92-9.14, *P* < 0.001) greater risk of developing T2DM when compared to those in the lower level. Highest IL-6 and TNF-*α* level groups were more likely to increase the risk of developing T2DM (OR = 1.66, 95% CI: 0.74-3.72, and OR = 1.51, 95% CI: 0.69-3.34, respectively). After adjusting for confounders in the multivariate analysis, a statistically increased risk of developing T2DM was obviously found in subjects with elevated CRP levels (OR = 4.02, 95% CI: 1.77-9.12, *P* = 0.001). Although there was no statistically significant association, a trend towards elevated risk of developing T2DM was observed in subjects with the highest TNF-*α* and IL-6 concentration ranges.

### 3.5. Combination Effects of Baseline Serum Inflammatory Marker Levels on the Risk of Developing T2DM

Since consideration of a single inflammatory marker alone may not show the statistical relationship with risk of developing T2DM, we therefore performed the combination analysis to evaluate the interaction effect in each inflammatory marker. The results of logistic regression for the 2-year T2DM progression are presented in Table 4. The association was obviously found in the synergistic interaction term between the high level of baseline CRP and IL-6; subjects with this combination had a substantially increased risk of T2DM by almost 6 times compared with the low-level group (OR = 5.96, 95% CI: 1.55-22.88, *P* = 0.009). The effects were still statistically significant after adjusting for confounders; the synergistic interaction value was 5.1 (OR = 5.11, 95% CI: 1.27-20.49, *P* = 0.021). Subjects with a combined elevation of baseline CRP and TNF-*α* levels indicated a significantly increased risk of developing T2DM (OR = 4.11, 95% CI: 1.10-15.33, *P* = 0.035) when compared with the low-level group in the adjusted analysis. Conversely, the combination of elevated levels of baseline IL-6 and TNF-*α* was not significantly associated with T2DM; however, subjects in this group showed a slight trend towards increased risk of developing T2DM (OR = 2.34, 95% CI: 0.79-6.97, *P* = 0.127). Furthermore, we performed an analysis of synergistic interaction among all 3 inflammatory markers. The effect of combined high CRP, IL-6, and TNF-*α* levels was found with a significantly increased risk to develop T2DM in the crude analysis (OR = 5.71, 95% CI: 1.07-30.63, *P* = 0.042), whereas this relationship was not rather strong after the adjustment of confounding factors.

### 3.6. The Association between Changes in Serum Inflammatory Marker Levels and the Risk of Developing T2DM

As displayed in Table 5, subjects with a consistently high level of CRP in 1 year had approximately 4 times (OR = 4.23, 95% CI: 1.54-11.59, and *P* = 0.005) greater risk of developing T2DM, compared to those with a consistently low level. After adjusting for confounders, high CRP levels remained a significant association with increased risk of developing T2DM (OR = 4.22, 95% CI: 1.45-12.31, *P* = 0.008). T2DM risk significantly increased in subjects with high IL-6 levels at both baseline and the first year of reexamination (OR = 4.14, 95% CI: 1.06-16.21), and elevated IL-6 levels for 1 year showed a trend towards increased risk of developing T2DM. However, these trends attenuated after the adjustment of confounding factors. Considering changes in TNF-*α* levels, the risk of developing T2DM after 2 years significantly increased by about 5 times in subjects with high TNF-*α* levels at the first year of reexamination, no matter if the levels were low or high at baseline. Moreover, a statistical significant effect was also found after adjusting for confounders (OR = 4.88, 95% CI: 1.01-23.49, *P* = 0.048 for low to high, and OR = 3.58, 95% CI: 1.02-12.55, *P* = 0.047 for high to high).

## 4. Discussion

In this retrospective cohort study, we primarily found the positive predictive power of inflammatory markers on blood glucose concentrations. The results further showed the evidence that the risk of developing T2DM was significantly increased among subjects with elevated baseline CRP levels, and this association was stronger in combination between high levels of baseline CRP and IL-6. Additionally, we also pointed that a notable association between one-year elevations of TNF-*α* levels and T2DM progression among the participants represents the Thai population in a rural area where nutrition transition occurs.

As increasing age, overweight, impaired glucose tolerance, and family history have been suggested to be major risk determinants for T2DM [2], older subjects in this study were more likely to develop T2DM than the younger group. Statistical outcome displayed baseline BMI, WC, WHR, and blood glucose values higher in the participants with T2DM progression. Although it did not reach statistical significance, the T2DM group indicated a trend towards more family members suffering from diabetes. These diabetes risk factors should be firstly emphasized for planning a disease prevention program in this cohort. In contrast, some well-known modifiable factors, including smoking, physical inactivity, and unhealthy food intake, were not found to have any association with the development of T2DM in the present study. These nonstatistically significant results were probably due to self-reported data depending on participants' memories.

Focusing on inflammatory markers, the difference of baseline CRP, IL-6, and TNF-*α* levels between the normal and T2DM progression groups was found in our study. This is consistent with previous findings which showed the positive correlation of inflammatory markers and blood glucose indices [10, 12, 29]. Moreover, a predictive analysis using multiple linear regression significantly revealed that CRP and IL-6 levels at baseline could be possible biomarkers for predicting blood glucose. This may imply their certain role in relation to the hyperglycemic state. Primary outcomes of baseline parameters in the present study provided a basic assumption regarding directionality in the association of inflammatory marker levels with glucose homeostasis.

As elevated CRP levels were suggested as an independent predictor for incident T2DM [5–9], a recent study conducted in the same cohort also showed the significant association of CRP levels and T2DM [12]; however, this cross-sectional analysis may not clearly describe that CRP is the actual supportive cause for the development of T2DM. In the present study, a four times higher risk of developing T2DM by baseline CRP could establish a temporal relationship, the time sequence between a factor and disease, among this cohort. As with other longitudinal analyses suggesting the effect of stable CRP levels during a long-term period [32, 33], our study also found the corresponding result which was constantly elevated for 1 year. These findings support the hypothesis that CRP may have indirect effect on insulin sensitivity and insulin production from pancreatic *β*-cells through the alteration of innate immune response due to elevated systemic inflammation [34]. High levels of CRP are also involved in the production of adhesion molecules, namely, E-selectin, ICAM-1, and VCAM-1, which play a role in vascular endothelial dysfunction, insulin transport reduction, and peripheral insulin resistance [35]. The positive relationship of CRP and T2DM progression probably relates to atherosclerosis development. However, our study completely excluded subjects with CVD at baseline examination, and new-onset CVD was not reported during the follow-up period.

Chronic low-grade inflammatory processes are initiated by the accumulated fat in adipocytes. While the activation of inflammation is constantly increasing, inflammatory cells, mainly macrophages, are recruited to infiltrate in adipose tissue. Macrophages take responsibility for producing and secreting inflammatory cytokines such as IL-6 and TNF-*α* [15, 16, 20]. Elevations in these cytokine levels have direct influence on insulin signal intensity, contributing to insulin resistance in the target cell. Impaired insulin signal transduction may be explained by a principal molecular mechanism, the serine phosphorylation of insulin receptor substrate 1 (IRS-1), which mediates insulin resistance through inhibitory effects on glucose transporter 4 (GLUT4) [36–38]. Indirect effects of IL-6 and TNF-*α* are also found by inducing CRP production through upregulation of transcription factors [39]. Thus, these inflammatory cytokines are involved in obesity-related T2DM. However, this study, as well as a previous study with the same cohort [12], reported weak positive correlations of anthropometric parameters and 2 cytokines. Moreover, there was a trend slightly towards increased risk of T2DM with baseline IL-6 and TNF-*α* levels in the highest tertile, while several studies have seen the elevated level of both cytokines as the risk of developing T2DM after adjusting for confounders [12, 40–43]. Nevertheless, there were unclear findings that the strength of relationship between IL-6 and T2DM in multivariate analysis was dramatically attenuated [9], and a nonstatistically significant result was found between TNF-*α* and T2DM risk [12]. Compared with CRP, IL-6 and TNF-*α* showed weaker and more inconsistent associations with T2DM development. Both of them probably have greater variability and no obvious cut point.

Interestingly, we performed further analysis which indicated the combined effects of the three inflammatory markers on the 2-year risk of developing T2DM. A combined analysis obviously showed a significant interaction between baseline CRP and IL-6. The synergistic interaction between high levels of baseline CRP and IL-6 had 5.1 times elevated risk of developing T2DM, which was a 110 percent increase compared with the high levels of CRP alone. Study subjects with combined high levels of CRP and TNF-*α* were found to have an increased risk of T2DM; however, this effect was not strong enough to be determined as synergism. Our findings also demonstrated an effect of combined consideration between IL-6 and TNF-*α*. This seemed to be an antagonistic interaction in the condition of high IL-6 and TNF-*α* levels. Likewise, interaction analysis of the cytokines in the EPIC-Potsdam study revealed that a combined elevation of IL-6 and TNF-*α* did not show a trend towards increased risk of developing T2DM [8]. These results suggested that the combination pattern of inflammatory cytokines may be related to the pathogenesis of T2DM. Our findings corresponded to the knowledge that inflammatory responses rely on several cytokines rather than on a single cytokine alone, and cytokines systematically operate in the form of a complex signaling network [35]. The influence of cytokines on insulin resistance may be considered with the combined elevation of various cytokines. Apart from IL-6 and TNF-*α*, other potential inflammatory cytokines, such as IL-1*β* and IL-8, have previously showed the effect of desensitization on insulin signaling via I*κ*B kinase *β* [44] and may be involved with poor glycemic control through activation of the Toll-like receptor 2 (TLR2) on B cells [45, 46].

In addition to cytokine levels at a single time, two consecutive examinations at the years 2013 and 2014 enable us to evaluate the relationship of IL-6 and TNF-*α* changes with the risk of T2DM. The consistent 1-year high of IL-6 and TNF-*α* levels was significantly associated with T2DM, although the risk attenuated after the adjustment for confounders. Moreover, a remarkable relationship was found between the risk of developing T2DM and increments of TNF-*α* level within 1 year. This result implies that insulin resistance probably occurs once TNF-*α* levels are initially risen at baseline and continuously elevated thereafter and may underline early detection of chronic low-grade inflammation levels in order to reduce the risk of developing T2DM. A longitudinal study with a longer follow-up period, more frequent examination, and larger sample size would be useful to clarify the effects of variation in inflammatory marker levels on T2DM progression.

## 5. Conclusions

Our 2-year retrospective analysis revealed that high baseline CRP levels were related with increased T2DM risk, and the impact was substantially greater when combined with other high inflammatory marker levels, particularly with IL-6. This study also showed strong relationship of 1-year TNF-*α* changes with T2DM progression. Thus, CRP and IL-6 could be an effective combination for identifying as a T2DM risk factor. A one-year TNF-*α* variation may initiate T2DM and play a role as a potential predictive marker for T2DM. These results substantially support the role of inflammatory markers in the pathogenesis of T2DM and emphasize that the identification of biomarkers for predicting T2DM will be beneficial to develop effective diabetes prevention and an early detection program. The limitation of this study should be addressed. First, we cannot control the assessment of independent or dependent variables, because a retrospective design must rely on the accuracy of collected data from others. Second, a retrospective study may lead to selection bias and information bias. Third, the present study is a short-term retrospective cohort design; while this enabled it to investigate the associations for a short period, it may not provide reasonable information on longitudinal relationships of this chronic disease. Fourth, our study was performed with small sample sizes. Therefore, a future long-term cohort study with larger sample sizes is needed to clarify the predictive ability of these inflammatory markers on the risk of developing T2DM in rural Thais.

## Figures and Tables

**Table 1:** Baseline characteristics of study subjects in longitudinal analysis based on progression to T2DM.

	Nonprogression group	Progression group	*P* value
Number	202	46	
Age (years)	45 (40-49)	47.5 (42-52)	0.035
Gender (male/female)	66/136	16/30	0.784
Current smoker (%)	40 (19.8%)	10 (21.7%)	0.956
Alcoholic drinking (%)	92 (45.5%)	18 (39.1%)	0.498
Lack of exercise (%)	54 (26.7%)	12 (26.1%)	0.614
Family history of diabetes (%)	71 (35.1%)	23 (50.0%)	0.061
BMI (kg/m^2^)	25.17 (22.11-27.85)	27.08 (22.37-31.11)	0.020
WC (cm)	83.00 (75.00-89.00)	89.25 (77.00-95.50)	0.003
WHR	0.87 (0.83-0.92)	0.90 (0.85-0.95)	0.014
Body fat (%)	30.65 (23.75-35.10)	33.00 (25.53-37.63)	0.058
Visceral fat (%)	8.50 (6.00-12.50)	11.00 (7.88-16.88)	0.008
Muscle (%)	25.45 (23.60, 30.40)	24.75 (22.60-29.50)	0.078
SBP (mmHg)	120 (111-129)	123 (114-130)	0.184
DBP (mmHg)	76 (70-81)	78 (73-86)	0.046
FBG (mg/dl)	90 (87-98)	103 (93-117)	<0.001
2-h BG (mg/dl)	113 (98-140)	173 (117-185)	<0.001
HbA1C (%)	5.3 (4.9-5.6)	5.6 (5.2-6.1)	<0.001
HOMA-IR	1.18 (0.80-1.59)	1.63 (1.10-2.68)	0.001
HOMA-*β*	66.17 (43.64-97.75)	56.66 (26.33-106.70)	0.158
Triglyceride (mg/dl)	120 (89-170)	139 (111-227)	0.017
Total cholesterol (mg/dl)	200 (173-222)	203 (178-225)	0.822
HDL-C (mg/dl)	52 (42-62)	45 (36-54)	0.005
LDL-C (mg/dl)	122 (96-149)	120 (93-143)	0.705
CRP (mg/dl)	1.39 (0.61-3.03)	2.81 (1.48-6.65)	0.001
IL-6 (pg/ml)	14.51 (4.31-248.93)	18.53 (5.25-732.45)	0.268
TNF-*α* (pg/ml)	53.80 (27.22-79.72)	62.28 (31.28-89.87)	0.272
Daily caloric intake (kcal/day)	2181.20 (1516.80-2900.81)	2071.50 (1581.53-3118.68)	0.910
Carbohydrate intake (% of DCI)	66.89 (60.30-72.18)	67.49 (61.10-73.95)	0.779
Protein intake (% of DCI)	11.03 (9.78-12.53)	10.90 (9.87-12.57)	0.906
Fat intake (% of DCI)	20.38 (15.27-25.90)	19.97 (15.00-24.83)	0.645
Total dietary GL (per day)	186.80 (125.80-279.97)	207.44 (132.48-248.02)	0.778

Data presented as median (interquartile range) or number (%). Abbreviations: BMI: body mass index; WC: waist circumference; WHR: waist-hip ratio; SBP: systolic blood pressure; DBP: diastolic blood pressure; FBG: fasting blood glucose; 2-h BG: 2-hour blood glucose after 75 g oral glucose tolerance test; HbA1c: hemoglobin A1c; HOMA-IR: homeostatic model assessment of insulin resistance; HOMA-*β*: homeostatic model assessment of *β*-cell function; HDL-C: high-density lipoprotein cholesterol; LDL-C: low-density lipoprotein cholesterol; CRP: C-reactive protein; IL-6: interleukin 6; TNF-*α*: tumor necrosis factor alpha; DCI: daily caloric intake; GL: glycemic load.

**Table 2:** Multiple regression analysis of blood glucose indices vs. 5 independent variables among 248 subjects at baseline.

Variables	FBG (mg/dl)^∗^		2-h BG (mg/dl)^∗∗^		HbA1C (%)^∗∗∗^	
	Standardized coefficients (*β*)	*P* value	Standardized coefficients (*β*)	*P* value	Standardized coefficients	*P* value
Age (years)	0.163	0.010	0.120	0.057	0.188	0.003
Sex	-0.129	0.044	0.155	0.015	0.079	0.215
CRP (mg/dl)	0.138	0.028	0.216	0.001	0.223	<0.001
IL-6 (pg/ml)	0.137	0.043	0.059	0.376	-0.054	0.420
TNF-*α* (pg/ml)	0.010	0.878	0.041	0.542	0.084	0.216

Abbreviations: FBG: fasting blood glucose; 2-h BG: 2-hour blood glucose after 75 g oral glucose tolerance test; HbA1c: hemoglobin A1c; CRP: C-reactive protein; IL-6: interleukin 6; TNF-*α*: tumor necrosis factor alpha. ^∗^*F* = 4.759 and *P* < 0.001 vs. 5 independents. ^∗∗^*F* = 4.800 and *P* < 0.001 vs. 5 independents. ^∗∗∗^*F* = 5.037 and *P* < 0.001 vs. 5 independents.

**Table 3:** Odds ratios (ORs) and 95% confidence intervals (CIs) for 2-year progression of type 2 diabetes according to baseline serum inflammatory marker levels.

Variables	Univariate analysis		Multivariate analysis	
	Crude OR (95% CI)	*P* value	Adjusted OR^∗^ (95% CI)	*P* value
CRP (mg/dl)				
Low level (<1.00)	1		1	
High level (≥1.00)	4.20 (1.92-9.14)	<0.001	4.02 (1.77-9.12)	0.001
IL-6 (pg/ml)				
1^st^ tertile (<5.93)	1		1	
2^nd^ tertile (5.93-85.65)	1.19 (0.53-2.68)	0.679	1.08 (0.46-2.52)	0.828
3^rd^ tertile (>85.65)	1.51 (0.69-3.34)	0.303	1.57 (0.68-3.59)	0.291
TNF-*α* (pg/ml)				
1^st^ tertile (<36.47)	1		1	
2^nd^ tertile (36.47-73.30)	1.41 (0.62-3.21)	0.408	1.35 (0.57-3.19)	0.489
3^rd^ tertile (>73.30)	1.66 (0.74-3.72)	0.215	1.63 (0.70-3.79)	0.260

Abbreviations: CRP: C-reactive protein; IL-6: interleukin 6; TNF-*α*: tumor necrosis factor alpha. ^∗^OR was adjusted for age, gender, WHR, VF, and TG.

**Table 4:** Odds ratios (ORs) and 95% confidence intervals (CIs) for 2-year progression of type 2 diabetes according to interaction between baseline serum CRP, IL-6, and TNF-*α* levels.

Variables	Univariate analysis		Multivariate analysis	
	Crude OR (95% CI)	*P* value	Adjusted OR^∗^ (95% CI)	*P* value
Interaction of CRP^a^ and IL-6^b^				
CRP (low)+IL-6 (low)	1		1	
CRP (low)+IL-6 (high)	2.28 (0.62-8.45)	0.218	1.78 (0.45-7.00)	0.412
CRP (high)+IL-6 (low)	2.65 (0.67-10.47)	0.164	2.01 (0.48-8.49)	0.340
CRP (high)+IL-6 (high)	5.96 (1.55-22.88)	0.009	5.11 (1.27-20.49)	0.021
Interaction of CRP and TNF-*α*^c^				
CRP (low)+TNF-*α* (low)	1		1	
CRP (low)+TNF-*α* (high)	1.61 (0.49-5.26)	0.429	1.51 (0.44-5.16)	0.509
CRP (high)+TNF-*α* (low)	1.23 (0.34-4.47)	0.756	1.17 (0.31-4.46)	0.822
CRP (high)+TNF-*α* (high)	4.05 (1.18-13.85)	0.026	4.11 (1.10-15.33)	0.035
Interaction of IL-6 and TNF-*α*				
IL-6 (low)+TNF-*α* (low)	1		1	
IL-6 (low)+TNF-*α* (high)	1.37 (0.55-3.40)	0.504	1.28 (0.50-3.32)	0.607
IL-6 (high)+TNF-*α* (low)	2.05 (0.44-9.47)	0.359	1.65 (0.33-8.42)	0.544
IL-6 (high)+TNF-*α* (high)	1.99 (0.71-5.58)	0.190	2.34 (0.79-6.97)	0.127
Interaction of CRP, IL-6 and TNF-*α*				
CRP (low)+IL-6 (low)+TNF-*α* (low)	1		1	
CRP (high)+IL-6 (high)+TNF-*α* (high)	5.71 (1.07-30.63)	0.042	4.77 (0.84-27.11)	0.078

Abbreviations: CRP: C-reactive protein; IL-6: interleukin 6; TNF-*α*: tumor necrosis factor alpha. ^∗^OR was adjusted for age, gender, WHR, VF, and TG. ^a^CRP (low): serum CRP < 1.00 mg/dl; CRP (high): serum CRP ≥ 1.00 mg/dl. ^b^IL-6 (low): serum IL-6 in 1^st^ tertile (<5.93 pg/ml); IL-6 (high): serum IL-6 in 3^rd^ tertile (>85.65 pg/ml). ^c^TNF-*α* (low): serum TNF-*α* in 1^st^ tertile (<36.47 pg/ml); TNF-*α* (high): serum TNF-*α* in 3^rd^ tertile (>73.30 pg/ml).

**Table 5:** Odds ratios (ORs) and 95% confidence intervals (CIs) for 2-year progression of type 2 diabetes according to 1-year changes of serum inflammatory marker levels.

Variables	Univariate analysis		Multivariate analysis	
	Crude OR (95% CI)	*P* value	Adjusted OR^∗^ (95% CI)	*P* value
1-year changes of serum CRP^a^				
Low (baseline)-low (1 year)	1		1	
Low (baseline)-high (1 year)	0.66 (0.17-2.59)	0.548	0.65 (0.16-2.60)	0.539
High (baseline)-high (1 year)	4.23 (1.54-11.59)	0.005	4.22 (1.45-12.31)	0.008
1 year changes of serum IL-6^b^				
Low (baseline)-low (1 year)	1		1	
Low (baseline)-high (1 year)	4.17 (0.88-19.82)	0.073	3.36 (0.67-16.94)	0.142
High (baseline)-high (1 year)	4.14 (1.06-16.21)	0.041	3.68 (0.91-14.90)	0.068
1-year changes of serum TNF-*α*^c^				
Low (baseline)-low (1 year)	1		1	
Low (baseline)-high (1 year)	5.28 (1.18-23.71)	0.030	4.88 (1.01-23.49)	0.048
High (baseline)-high (1 year)	4.12 (1.22-13.93)	0.023	3.58 (1.02-12.55)	0.047

Abbreviations: CRP: C-reactive protein; IL-6; interleukin 6; TNF-*α*: tumor necrosis factor alpha. ^∗^OR was adjusted for age, gender, WHR, VF, and TG. ^a^Low (baseline) and low (1 year): serum CRP < 1.00 mg/dl; high (baseline) and high (1 year): serum CRP ≥ 1.00 mg/dl. ^b^Low (baseline): serum IL-6 in 1^st^ tertile at baseline (<5.93 pg/ml); high (baseline): serum IL-6 in 3^rd^ tertile at baseline (>85.65 pg/ml); low (1 year): serum IL-6 in 1^st^ tertile at 1 year of follow-up (<15.14 pg/ml); high (1 year): serum IL-6 in 3^rd^ tertile at 1 year of follow-up (>75.67 pg/ml). ^c^Low (baseline): serum TNF-*α* in 1st tertile at baseline (<36.47 pg/ml); high (baseline): serum TNF-*α* in 3^rd^ tertile at baseline (>73.30 pg/ml); low (1 year): serum TNF-*α* in 1^st^ tertile at 1 year of follow-up (<33.78 pg/ml); high (1 year): serum TNF-*α* in 3^rd^ tertile at 1 year of follow-up (>75.61 pg/ml).

## Data Availability

The data used to support the findings of this study are available from the corresponding author upon request.
